# Beyond the Locked Door: Hikikomori Through a Clinical Lens

**DOI:** 10.7759/cureus.78600

**Published:** 2025-02-06

**Authors:** Luís Paulino Ferreira, Margarida Magalhaes, Maria Miguel Figueiredo, Ema Conde

**Affiliations:** 1 Psychiatry and Mental Health, Unidade Local de Saúde da Arrábida, Setúbal, PRT; 2 Psychiatry and Mental Health, Unidade Local de Saúde de Loures-Odivelas, Loures, PRT

**Keywords:** cultural mental health, digital media and isolation, hikikomori syndrome, psychiatric comorbidities, social withdrawal

## Abstract

Hikikomori, first identified in Japan, refers to individuals who withdraw from society and confine themselves to their homes, often remaining isolated in their bedrooms. Those affected typically disengage from education, employment, and daily activities, leading to significant functional impairment. While initially considered a culture-bound syndrome, growing evidence in recent decades suggests that hikikomori is a global phenomenon. Individuals with this condition often come into contact with healthcare systems through legal interventions. Here, we present two cases of Portuguese males with hikikomori syndrome who have committed sexual crimes.

## Introduction

Hikikomori is a Japanese concept that emerged in the late 20th century, combining the characters “to pull back” (hiku) and “to seclude oneself” (komoru) [1]. The term gained recognition in 1988 when psychiatrist Tamaki Saitō identified a growing number of adolescents exhibiting traits such as social withdrawal, lethargy, prolonged isolation lasting months or years, and neurotic-type psychiatric symptoms [2,3]. Initially regarded as a culture-bound syndrome unique to Japan, hikikomori was primarily associated with adolescents and young adults who withdrew into their parents’ homes, disengaging from social roles such as education and employment [4].

Hikikomori is a progressive condition characterized by ongoing functional decline and an absence of efforts to reintegrate into society. If left untreated, it can persist for years or even decades, leading to profound psychological distress and functional impairment [1,3]. Over time, similar cases have been documented in other Asian countries and, increasingly, in Western nations, suggesting that hikikomori may reflect a universal response to cultural and societal stressors rather than being confined to Japanese society [3,5].

Between 2002 and 2006, the World Mental Health Japan Survey Group conducted an epidemiological study estimating a lifetime prevalence of approximately 1.2% [3]. The condition was found to be four times more common in males than in females (1.8% vs. 0.4%), with most cases emerging during adolescence or young adulthood (ages 10-29: 82.5%) [2,3].

Epidemiological studies on hikikomori outside Japan remain scarce. However, an international investigation by Kato et al. [6] presented case vignettes of hikikomori to 247 psychiatrists and psychiatric trainees, finding no significant differences in estimated prevalence rates between Japan and other countries.

Here, we present two cases of males, aged 31 and 34, who remained confined to their homes for several years and came into contact with healthcare services following legal intervention for sexual offenses.

## Case presentation

Case 1

A 31-year-old male with a medical history of Hashimoto’s thyroiditis and obesity was referred for psychiatric evaluation by his primary care physician. The referral was accompanied by a recent analytical assessment, including blood tests for complete blood count and thyroid function, all of which were within the reference range.

The patient attended the evaluation reluctantly, stating, “I felt compelled to go because of the legal proceedings.” He had been convicted of possession of child pornography and sentenced to eight months in prison, suspended for one year.

His history revealed significant academic and social difficulties. He had failed three academic years before completing high school at the age of 21. He had been unemployed for over three years and lived exclusively with his parents. Since the age of 18, he had demonstrated a lack of initiative, particularly in seeking employment, and had confined himself primarily to his home. His daily activities consisted of watching television and playing computer games, with no other hobbies or structured activities.

Socially, the patient described himself as shy and expressed discomfort in public spaces, believing that others judged him negatively. He reported losing contact with a small group of adolescent friends and had never formed romantic or sexual relationships. He explicitly denied consuming both paraphilic and non-paraphilic pornography beyond that for which he was convicted, as well as any history of substance use.

Mental Status Examination

The patient presented with a withdrawn posture and a minimally groomed appearance. He was alert and oriented but avoidant in eye contact. His attention was capturable and fixable. He exhibited a euthymic mood with poorly modulated affect and latent anger. His speech was sparse but coherent, with normal rate, volume, and syntax. His thought process was logical, and there were no perceptual disturbances. Sleep disturbances included delayed onset, while his appetite remained normal. He expressed insight into his social and interpersonal deficits but lacked the motivation to address them.

Clinical Course

During subsequent consultations, the patient showed no motivation to engage in treatment or adhere to the therapeutic plan. He did not take the prescribed medications (sertraline 50 mg/day and trazodone 150 mg/day) and consistently failed to attend psychotherapy sessions. Based on the clinical evaluation, he was diagnosed with avoidant personality traits.

Case 2

A 34-year-old male attended psychiatric consultations after being sentenced to one year and six months in prison, suspended for one year, for the crime of child pornography. The patient had dropped out of school in the 10th grade and worked with his father from the ages of 14-17, the longest job he had held. Afterward, he had several undifferentiated short-term jobs and had been unemployed for the past seven years. The patient had always lived with his parents and sister, and for the past four years, he had rarely left his room, even during family gatherings.

The patient stated that he does not leave the house due to “laziness, not wanting to care about the world, and being angry with myself and the world” (sic). He spends his day watching television and playing computer games. The patient described himself as someone who had difficulty making friends, with most of his friends being virtual. He also reported having low frustration tolerance, which had led to episodes of hetero-aggressiveness directed at objects. The patient’s first sexual experience occurred with a sex worker at approximately 16 years of age. He had two romantic relationships, lasting five years and six months, respectively. His last romantic relationship had been an online one. He had not had a sexual relationship for approximately four years. The patient denied having pedophilic fantasies but frequently fantasized about older women and trans women who had not undergone complete transition, retaining male genitalia.

He had experienced two episodes of depression in the past, at ages 28 and 30. During those times, antidepressants were prescribed, but he chose not to take the medication. Since being confined to his room, he has begun using cannabinoids daily. Additionally, he had experimented with cocaine multiple times around the age of 20.

Mental Status Examination

The patient was alert and oriented in all references, with avoidant contact. His attention was capturable and fixable. His mood was euthymic, but his emotions and affect were poorly modulated. His speech was circumstantial, with no alterations in rate, volume, semantics, prosody, or syntax. His thought process showed no alterations in course, form, or possession. No changes in sensory perception were noted. Sleep and appetite were normal. The patient exhibited limited insight into his social and interpersonal deficits.

Clinical Course

During subsequent consultations, the patient showed no motivation to engage in treatment or adhere to the therapeutic plan. Based on the clinical evaluation, he was diagnosed with avoidant personality traits.

Table 1 presents a comparative summary of the key clinical and demographic aspects of both cases.

**Table 1:** Comparison of Case 1 and Case 2

Characteristic	Case 1	Case 2
Age	31 years	34 years
Previous medical history	Hashimoto’s thyroiditis and obesity	None
Legal background	Eight-month prison sentence, suspended for one year, for possession of child pornography	One year and six months prison sentence, suspended for one year, for child pornography
Education and labor activity	Repeated three grades; completed high school at 21 years; unemployed for over three years; no initiative to seek jobs	Dropped out in 10th grade; unemployed for seven years; had undifferentiated short-term jobs
Social functioning	Always lived with his parents; avoided public spaces; had no close friendships; solitary hobbies (watching television and playing computer games)	Lives with parents and sister; rarely leaves room; few friends, mostly virtual; solitary hobbies (watching television and playing computer games)
Affective/sexual history	No romantic relationships or sexual experiences	First sexual experience with a sex worker at 16, two romantic relationships (one online); no relationships in the last four years
Paraphilic interests	Denies any paraphilic interests beyond conviction	Denies pedophilic fantasies; frequently fantasizes about older women and trans women retaining male genitalia
Substance use	Denies psychoactive substance use	Daily cannabinoid use; past use of cocaine
Mental status examination	Withdrawn posture; minimally groomed appearance; avoidant contact; euthymic mood with poorly modulated affect and latent anger; delayed sleep onset; no alterations in thought or sensory perception	Avoidant contact; euthymic mood; poorly modulated affect; circumstantial speech; normal sleep and appetite; no alterations in thought or sensory perception
Insight and motivation for change	Critical of social and interpersonal deficits, but no motivation for change; non-adherent to treatment	Limited insight into social and interpersonal deficits; no motivation for change; non-adherent to treatment
Psychiatric history	None	Two episodes of depression at ages 28 and 30, without therapeutic adherence
Diagnosis	Avoidant personality traits	Avoidant personality traits

## Discussion

The criteria for diagnosing hikikomori have evolved over the past few decades. Although there is no universal consensus, the most widely accepted definition includes three key elements, as outlined by Kato et al. [1]: (a) social isolation within the home, involving physical withdrawal to one’s place of residence, lack of active participation in educational and work environments, and limited engagement in social relationships; (b) the duration of social isolation must be at least six months; and (c) significant functional impairment or distress is associated with the isolation.

In the cases presented, both patients met all the diagnostic criteria for hikikomori. Neither patient participated in social activities such as attending work or school and maintained only minimal interpersonal relationships. Regarding severity, individuals who remain confined to their rooms with limited interaction, even with cohabiting family members, are considered to have severe cases [7]. Both patients were entirely dependent on family support for housing and financial needs.

The age of onset in both cases was consistent with existing literature, with most cases emerging during adolescence or early adulthood [1]. Case 1 had repeated failures to progress to the next grade, while Case 2 dropped out of school, mirroring findings that individuals with hikikomori often abandon education, exhibit high absenteeism, and face multiple academic failures [2]. Prolonged absenteeism from school is often considered an early indicator of the syndrome [4].

Hikikomori is more prevalent in individuals with mental disorders such as depressive disorder, social anxiety disorder, schizophrenia, autism spectrum disorder, and avoidant personality disorder [8,9]. Both patients in this case report were diagnosed with avoidant personality traits. In a case-control study, Muris et al. [4] found a significant link between addiction and hikikomori, with substances such as alcohol, cocaine, amphetamines, and cannabis being commonly abused - substances that were also reported in Case 2, who had a history of cannabis use.

The internet and digital media play a complex role in the lives of individuals with hikikomori. On one hand, these platforms can perpetuate isolation, with hikikomori being linked to problematic internet use and video game addiction, especially in more severe cases of social withdrawal [10,11]. On the other hand, some literature suggests that digital platforms can serve as a “social lifeline,” allowing easier communication for those who struggle with face-to-face interactions due to advanced social skill deficits [12]. Both patients communicated predominantly through virtual platforms. However, in these cases, it is difficult to identify any positive effects of internet use, particularly considering the sexual crimes committed.

The sexuality of individuals with hikikomori remains an underexplored area in the literature. Perrota and Piccininno [2] reported that 94 out of 98 patients experienced difficulties in accepting their sexual identity, manifesting as avoidant, hyposexual, or significantly dysfunctional sexual behaviors, including tendencies toward atypical or deviant sexual patterns. While Case 1 denied using the internet to explore his sexuality, the commission of the sexual offense through this medium suggests otherwise. Notably, no studies have established a direct correlation between hikikomori and the commission of sexual offenses.

Patients with hikikomori typically do not seek treatment voluntarily [3]. Therefore, family interventions are crucial, particularly when the individual resides with their family [1,3]. Another potential route for engagement is through home visits by healthcare professionals such as physicians, nurses, psychologists, and social workers [1]. When psychiatric comorbidities are present, psychotropic medications may be indicated. On a broader scale, psychotherapy is considered an effective intervention for hikikomori [1]. Specifically, psychodynamic therapies, whether delivered individually or in groups, have proven particularly helpful in addressing interpersonal difficulties. These therapeutic approaches are especially effective in improving family dynamics and fostering healthier interactions with peers in academic or workplace settings [1,3].

## Conclusions

Hikikomori is a complex and often refractory condition that results in significant suffering for affected individuals and their families. This case report highlights the challenges of managing hikikomori in the context of associated sexual offenses, avoidant personality traits, and comorbid psychiatric conditions, including problematic internet use and substance dependence. Crucially, hikikomori should not be regarded as a distinct psychiatric diagnosis, but rather as a broad category under which multiple psychiatric disorders may coexist. This perspective calls for a nuanced, individualized approach to each case, focusing on the underlying psychopathology. The cases presented emphasize the need for a multidisciplinary approach to managing hikikomori, addressing its psychological, social, and familial dimensions. Given the scarcity of literature on sexuality and legal issues in hikikomori, this article aims to stimulate further research to better understand these aspects and develop more targeted therapeutic strategies. By raising awareness, we hope to contribute to the advancement of care for individuals living with this profoundly isolating condition.

Despite its contributions, this case report has certain limitations. The small sample size of only two cases restricts the generalizability of the findings. Additionally, the lack of standardized psychometric assessments limits the validation of personality traits and cognitive function. A more comprehensive exploration of differential diagnoses, psychiatric family history, and psychomotor development could have provided further clinical insight. Future studies should aim to address these gaps through larger, methodologically rigorous investigations.
